# Methodological reflections from a research project on the mental health of Black youth

**DOI:** 10.24095/hpcdp.45.4.05

**Published:** 2025-04

**Authors:** Bukola Salami, Jordana Salma, Benjamin Denga, Aloysius Maduforo, Dominic A. Alaazi

**Affiliations:** 1 Department of Community Health Sciences, University of Calgary, Calgary, Alberta, Canada; 2 Faculty of Nursing, University of Alberta, Edmonton, Alberta, Canada; 3 NorQuest College, Edmonton, Alberta, Canada; 4 Faculty of Health Sciences, University of Western Ontario, London, Ontario, Canada

**Keywords:** methodology and research, mental health, adolescent health, Black youths, participatory

## Abstract

The aim of this study was to provide an illustrative example of how researchers can effectively engage Black youth using a culturally responsive, participatory action research (PAR) approach. We aimed to examine the mental health needs of Black youth and identify culturally relevant strategies to increase access to and uptake of mental health services. The study took a PAR approach to foster maximum inclusion of youth in the research process. We collected data in two phases: (1) individual interviews with 30 youths; and (2) monthly conversation cafs over a four-month period with 99 youth participants. We recruited youth participants through the Africa Centre Youth Empowerment Group in Alberta, at a soccer tournament hosted by Africa Centre and through affiliated social networks, and established a youth advisory group that met quarterly and assisted with data collection, data analysis and dissemination. We shared our findings at a community engagement session for stakeholders. The study provided space for youth to share their experiences and to imagine solutions to their mental health difficulties; it also allowed us to conduct research that carefully integrated the perspectives of those most affected by the study’s policy and practice recommendations. This project is an important case example that demonstrates promising practices and accessible methods across the data collection cycle, as well as the key ingredients and mechanisms that support culturally responsive practice.

**Highlights**

- This study examined the mental health needs of Black youth in Alberta.
- The study undertook a youth empowerment model situated within a postcolonial feminist paradigm to understand the mental health experiences and needs of Black youth.
- Researchers identified culturally relevant and effective approaches to improving access to and uptake of mental health services while capitalizing on the agency of this population.

## Introduction

Participatory research with youth is a transformative approach that positions youth as active co-researchers, enhancing their inclusion, empowerment and equitable decision-making throughout the research process.^1^,^2^ However, despite its potential, there has been limited research examining how to effectively conduct culturally responsive research with Black populations, particularly African, Black and Caribbean youth in Canada and around the world.^3^,^4^ This gap is evident in the scarcity of studies that prioritize culturally appropriate methods and address the unique social, structural and systemic barriers these youth face, including racism, discrimination and marginalization.^5^,^6^ As noted by Bailey et al., researchers often encounter difficulties in building trust with Black communities, exacerbating challenges in fostering meaningful engagement and generating impactful findings that resonate with the community.^7^

A culturally responsive and equitable approach to youth research requires thoughtful incorporation of practices that address power imbalances and the historical mistrust between Black communities and researchers.^4^ For example, collaborating with community gatekeepers, employing inclusive data collection strategies and ensuring Black leadership within research teams are critical to enhancing research acceptability and fostering local ownership of outcomes.^8^ Moreover, while this project focussed on African, Black and Caribbean youth, all participants self-identified as Black. Therefore, the term “Black youth” will be used interchangeably with “African, Black and Caribbean youth” to reflect participants’ self-identifications.

This paper offers an illustrative example of youth-led participatory action research (PAR) that successfully engaged Black youths in Alberta, Canada, to explore and address their mental health needs. Drawing on a postcolonial feminist empowerment model, this study integrated culturally responsive practices such as involving youth co-leadership, creating supportive research environments and ensuring the inclusion of diverse voices within the research process.^4^,^9^ By emphasizing the engagement of Black youth as co-researchers, the project not only promoted inclusivity but also fostered valuable insights into culturally appropriate and impactful research practices. 

These strategies are essential for researchers looking to ethically and meaningfully engage with Black youth populations.7 Studies suggest that African, Black and Caribbean populations, including youth, may experience higher rates of mental health problems compared to the general Canadian population.^10^-^12^

Despite this evidence, we are unaware of any Canadian study prior to the conceptualization of this research in 2017 that has provided a basis for action that could inform the efforts of African, Black and Caribbean youth to improve their mental health outcomes. Indeed, we conducted interviews with 57 African immigrant parents, community leaders, service providers and policy makers on parenting and mental health promotion practices; these individuals collectively expressed a strong need for research that will provide useful interventions to promote the mental health of African immigrant children and youth.^13^-^15^

Furthermore, our preliminary consultations with youths called for collective action among African, Black and Caribbean immigrant youth while acknowledging the vital influences of race and colonialism on their experiences. Thus, our study focussed on diverse generation (first, second, third, etc.) immigrant youths as well as those who migrated through various streams (immigrants, refugees and international students). While we recognize the heterogeneity of this group (i.e. African, Black and Caribbean youth, as well as refugees, immigrants and international students), we are also following the lead of youths consulted prior to our study. During our consultations, the youths emphasized the importance of focussing on the collective identity of African, Black and Caribbean youth rather than focussing on African, Black or Caribbean communities in isolation. Our definition of our population was thus informed by our consultations; we nonetheless maintained space for the analysis of subgroups. 

Overall, our project aimed to improve the mental health outcomes of African, Black and Caribbean youth in Alberta. This paper is an important case example that demonstrates promising practices and accessible methods across the data collection cycle, as well as the key ingredients and mechanisms that support culturally responsive practice.

## Purpose and research questions

The aim of this paper was to provide an illustrative example of how researchers can effectively engage African, Black and Caribbean youth in Canada using a PAR approach. By leveraging postcolonial feminist theory, the paper offers insights into culturally responsive and ethical research practices that promote meaningful collaboration and empowerment of Black youth as co-researchers, addressing systemic barriers such as racism and marginalization in the research process. From the outset, the PAR approach was employed to co-develop the project’s research questions in partnership with Black youth. Through early consultations with Black youths (described later in this article), the research questions were collaboratively crafted and subsequently refined to ensure alignment with funder priorities. The final research questions guiding this study were as follows:

- What are the mental health needs of African, Black and Caribbean youth in Alberta?
- What are the barriers to access to and use of mental health services for African, Black and Caribbean youth in Alberta?
- What are culturally relevant and effective approaches to increasing access to and uptake of mental health supports by African, Black and Caribbean youth in Alberta?
- What potential exists to mobilize African, Black and Caribbean youth to improve mental health outcomes and/or to build resilience and capitalize on their agency?

The outcomes of the project, addressing these questions, have been documented and shared in multiple peer-reviewed journal articles.^3^,^8^

## Methods

***Ethics approval***

This project received approval from the University of Alberta Research Ethics Board 1, # Pro00079877.

***Theoretical framework—postcolonial feminist empowerment model***

We used a youth empowerment model situated within a postcolonial feminist paradigm to understand the mental health experiences and needs of African, Black and Caribbean youth. The postcolonial feminist paradigm is a critical framework that draws on the intersections of postcolonial theory and feminist thought to examine how colonial histories, power structures and imperialist legacies continue to shape gender, race and class inequalities, particularly for marginalized groups in formerly colonized societies.^16^ It recognizes that colonialism did not only impose political and economic domination but also cultural and social hierarchies that persist today.^16^

Within this framework, gender is not an isolated factor but is deeply intertwined with race, class and other social categories, making it essential to address the unique experiences of marginalized populations,^17^ such as African, Black and Caribbean youth in Canada. Postcolonial feminism critiques traditional Western feminist frameworks for universalizing women’s experiences and ignoring the diverse struggles faced by women in the Global South. It rejects the notion of a singular “Third World Woman” as a monolithic subject of oppression, emphasizing instead the importance of understanding local contexts and the various forms of resistance employed by marginalized groups.^18^ In this way, the postcolonial feminist paradigm offers a more nuanced understanding of the unique social and structural barriers faced by Black youth, particularly the ways in which racism, systemic marginalization and colonial legacies intersect to affect their mental health and well-being.^4^,^16^

This paradigm is particularly well-suited for this study because it aligns with the PAR methodology, which seeks to challenge traditional power dynamics by actively involving youth as co-researchers. By positioning Black youth as experts of their own experiences, the postcolonial feminist approach not only acknowledges the historical and systemic oppressions they face but also values their agency in addressing these issues.^19^ This framework supports the ethical engagement of marginalized communities and fosters the development of culturally responsive and community-driven solutions to the mental health challenges these youth encounter. Therefore, the postcolonial feminist paradigm provides a robust theoretical lens to critically explore the intersections of race, gender and colonial history, making it the ideal model to address the research questions posed in this study.

Empowerment is defined as gaining control of one’s life through active participation, with an emphasis on creating and building awareness of and engagement with one’s environment.^20^ This empowerment model guides youth empowerment processes and outcomes in research by creating safe and supportive spaces, ensuring meaningful participation, equalizing power dynamics between adults and youth, providing opportunities for personal and community development and encouraging critical reflection on broader processes and structures that shape their lives.^21^ These five dimensions guided the research design to ensure adherence to participatory and community-driven principles. 

By creating a safe and supportive environment and encouraging meaningful participation, we acknowledged the different histories and impacts of social, economic and political marginalization experienced by African, Black and Caribbean communities.^5^,^6^ Providing spaces for critical reflection and community engagement allowed youths to understand the forces that influence their lives, articulate their experiences and recognize their capacity for creating change.^22^ By using an empowerment model, we made explicit our commitment to providing a space for youths to use their voices and to simultaneously recognize their strengths and capacity for resilience as well as the oppressive structures and narratives that hinder agency. 

***Youth-led participatory action research***

PAR is a power-equalizing, collaborative research approach that sees community members as partners in the research process and experts on the issues of concern in their lives.^23^,^24^ This methodology is based on principles of shared leadership, collaborative decision-making and researcher–community trust building with the end aim of creating sustainable, action-oriented research outcomes. PAR approaches that genuinely incorporate youth as equitable decision-makers and collaborators throughout the research process are rare, despite ample evidence of the positive outcomes.^1^ We see youth-led PAR as a valuable approach that allows for increased youth empowerment and sociopolitical engagement and will lead to improved understanding of the mental health needs of youth in ways that can inform policy and community health initiatives.^2^,^9^,^25^ We engaged in youth-led PAR with African, Black and Caribbean youths to better understand their experiences with mental health, their perceived mental health needs, barriers to meeting these needs and culturally appropriate strategies to improve access to and uptake of mental health services. 

**Consultation with youths**

Before this project began, the first author and principal investigator (BS) was a member of Africa Centre, a major service provider for Africans in Alberta. During one of the board meetings, she became aware that the youths had no resources to address issues that were of concern to them. She thus arranged to meet with the youth leader and subsequently with eight African, Black and Caribbean youths of the Africa Centre Youth Engagement Group (YEG). The youths identified a crucial need to improve mental health outcomes within the community and, with the mentorship of the principal investigator (PI), who is herself a young African immigrant, devised a plan of action to address mental health concerns. 

First, they identified a need for data to inform future programs and strategies around mental health. Second, they opted for participatory research methodology as a viable strategy to enhance mental health outcomes. Third, they stressed the need to shed light on issues of discrimination rooted in colonial histories that mediate their experience; they also wanted an intersectional approach to this postcolonial perspective in exploring their mental health concerns. Fourth, the youths wanted to involve African, Black and Caribbean youth, emphasizing their collective, racialized identity. 

Furthermore, with the help of the PI, the youths developed a five-year plan and an Alberta-wide strategy to improve their mental health, the first step of which was to actively participate in the application for a grant to collect data on African, Black and Caribbean youth mental health. The youth leader was a co-investigator on the grant proposal and was involved in its development.

**Youth advisory committee**

Upon receiving the funding, the PI communicated with the youth group. However, the PI went on a one-year maternity leave, during which time the project was suspended and the leadership of the youth group changed. Upon the PI’s return, a meeting was arranged with the new leadership and advertised to recruit advisory committee members. Ten youths indicated interest in being on the advisory committee and attended the first meeting. Advisory committee members were all female or nonbinary, reflecting the composition of the YEG’s Black youth empowerment group (“The Come Up”). 

Advisory committee members met three times throughout the research process. Sub-teams of members met with the PI and one of the two PhD student research assistants (DA) assigned to the project an additional six times. The youths also met with each other additional times and maintained a WhatsApp group for communication among themselves. This was interpreted as a strategy for them to negotiate power relations with the PI who, although Black, was older (late thirties). 

The role of the advisory committee members included advising on data collection procedures, on delivery of conversation cafs and interviews and on knowledge translation and dissemination. Before the initial meeting, we presented the youths with our ethics documents and our preliminary interview guide. The youths commented on and made recommendations for the interview guide, including adding questions on preferences for healthcare provider (as it relates to race concordant health service provision). Youths also emphasized their desire to be actively involved in the project, especially in the collection and analysis of data. 

We thus hired youths (at approximately $20/h) who were interested in actively being involved as research assistants (seven youths). Advisory committee members who were not research assistants (three youths) were also paid an honorarium totalling an equivalent amount. The advisory committee members who were hired as research assistants took more active roles in interviewing participants, organizing and leading conversation cafs, developing the initial conversation caf guide, facilitating participant recruitment, analyzing data and co-authoring reports and publications. 

We infused a number of strategies to build the capacity of these youths. Prior to data collection, all research personnel received four hours of training on qualitative research and completed a research ethics training module. Research assistants involved in data analysis completed one day of training on the use of NVivo qualitative data analysis software and individual mentorship by a PhD student and research coordinator skilled in the use of NVivo. Throughout the research process, the research assistants received close mentorship from two PhD student research coordinators and the PI. They also met with the project lead to discuss their plans before each conversation caf. 

***Data collection and analysis***

Data collection and analysis occurred between August 2019 and February 2020 in two phases: Phase 1 involved interviews with African, Black and Caribbean youths, while Phase 2 involved conversation cafs with youths.

**Phase 1: interviews**

We conducted 30 individual interviews with youths to obtain an in-depth understanding of their mental health experiences. Upon obtaining ethics approval, we recruited youths through the Africa Centre YEG, which is the Africa Centre’s youth collective and represents diverse African and Afro-Caribbean communities. We also recruited youths through the personal networks of our research assistants and advisory committee members. During our recruitment, we determined that males were underrepresented in our sample, likely due to the all-female, nonbinary composition of our advisory committee. To increase the gender diversity in our sample, we attended the All Africa Soccer Tournament and Festival (hosted by Africa Centre in Edmonton, Alberta) to recruit more male participants. All study participants were of African, Black or Caribbean heritage, aged between 16 and 30 and fluent in English. We strived for maximum variation sampling by recruiting participants with diverse immigration backgrounds, genders, countries of origin and religious backgrounds. 

Individual interviews lasted around one hour, were audio-recorded (to be later transcribed verbatim by an experienced transcriptionist) and were conducted in person at a location that was comfortable for participants. The interviews were all one-on-one interviews, except for one participant with significant mental health challenges who brought his sister to the interview for support. Individual interviews included a sociodemographic questionnaire and semistructured interview questions centred on personal mental health experiences, perceptions around available support systems, perceived barriers to mental health, culturally appropriate and effective strategies to improve access to and uptake of mental health services, and implications for research, policy and practice. 

The interviews were conducted by the youth research assistants or one of the PhD students, who wrote reflexive notes at the end of each interview. The reflexive notes summarized the interview, identified any information that might not be obvious in interview audio recording (such as nonverbal cues) and discussed how their positionality may have affected the interview process. Participants received $10 for transportation and a $25 honorarium for participating in the study.

**Phase 2: conversation cafs**

Conversation cafs effectively fostered youth engagement and dialogue because youths could take the lead in identifying issues of concern to them and in explaining the impact these issues have on their lives. Conducted monthly over four months, these three-hour conversation cafs constituted a crucial data source for our study. Each caf included 30 to 50 youths. Each conversation caf began with a 30-minute meal and social time for youths, followed by a guest speaker who provided a 30-minute presentation related to mental health. The meal was often African or Caribbean and always bought from a Black vendor. We ensured vegetarian options were available and that all meals were halal. 

Guest speakers were chosen based on the results of previous interviews and on the advice of the youth advisory committee members. Upon reflecting on the data from Phase 1, youth research assistants and advisory committee members met to determine conversation caf topics. The first conversation caf was on mental health promotion. The youth research assistants wanted to focus on mental health promotion, as it was the beginning of the semester. The speaker was a Black PhD-prepared therapist and community leader. The second conversation caf focussed on intergenerational relations. The session was led by a Black associate professor of counselling psychology. Following up on the presentation, the youth research assistants asked participants questions to spark conversations related to intergenerational relations (as detailed below). 

The third conversation caf focussed on intersectionality and mental health to address the needs of Black 2SLGBTQI+ populations. The conversation caf had a panel presentation by a Black 2SLGBTQI+ activist and leader, a non-Black 2SLGBTQI+ therapist and a Black female community leader who was also a social worker. The fourth conversation caf focussed on mental health policy and practice. The PI gave a brief presentation. The youths then led small-group discussions on policy implications. The research assistants also asked the youths to think through what an ideal mental health clinic should look like (these data were later used to support an application for the creation of a mental health clinic). 

Youths had around 1.5 hours for focus groups in breakout sessions with a semistructured interview guide. The interview guides were developed by the youth research assistants and shared with the PI prior to the study. The interview guide reflected the perspective of youths on their lived experiences. We had planned to audio-record the sessions, but the youths strongly advised against this; therefore, we instead took notes during the sessions. Upon completion of the breakout sessions, the youths reconvened in a bigger session for 30 minutes to report back and discuss strategies for action; detailed reflexive field notes were also collected during this portion. Youths received $10 for transportation-related costs.

The following are examples of conversation caf questions on intergenerational relationships and mental health:

- Your parents are a few of many immigrants who have escaped civil war, persecution and other traumatic events. You notice signs of PTSD in one of your parents and whenever you try to hint at it, they are in denial. How would you approach them about this? How would you wish they would respond?
- You decide that you do not want to attend university, but wish to pursue a different career (art, music, entrepreneurship). How do you think your parents will react? How comfortable are you sharing this with your parents?
- Your child comes home from school and tells you that they wish they had lighter skin. How would you respond to your child and why do you think they might feel this way?
- You approach your parents and try to explain to them that you are depressed. They tell you that you have food, clothes and a house to live in and have no reason to be depressed. How does this make you feel and why do you think they respond this way?
- It has been two years since you and your family immigrated to Canada. Before leaving to attend a cultural event, your child tells you they do not want to go because they do not claim your culture/ethnicity anymore. Where might your child’s feelings rise from? How do you respond?
- You are worried that your sibling may be contemplating suicide and, when you tell your parents, they tell you they are probably just experiencing stress and to pray for them. How do you respond to this?

***Data management***

Data were stored on a secured, internal shared drive at the University of Alberta Faculty of Nursing. Only the seven research assistants, two PhD student research coordinators and PI had access to the shared drive and the data. A central concern was issues related to confidentiality, especially given the small community in which we work. Thus, we developed our drive in such a way that the youth research assistants only had access to data from the interviews they conducted (usually 3–8 interviews per youth). The two PhD students (both of whom were Black males) and the PI had access to all data. However, at the data analysis stage, the youth research assistants indicated interest in analyzing the data themselves. Thus, we provided two youth research assistants with access to all data. The two youths trained in the use of NVivo data analysis software were mentored by a senior PhD student who has experience providing training in data analysis to graduate and undergraduate students. On the first two days, the youths analyzed the data in the presence of the graduate student to help with any challenges that arose.

***Data analysis***

Thematic data analysis was used to identify relevant patterns and themes emerging from the data. Thematic analysis allowed us to identify and analyze patterns of data while situating these patterns within the broader context of their occurrence.^26^ First, the research assistants and PI read the interview transcripts multiple times and developed a preliminary coding tree. Two youth research assistants then coded the data. These codes were expanded and condensed based on emerging data. The codes were then translated into themes. The themes were compared and contextualized with the notes from cafs and field notes prior to writing the results of the study. Thus, the steps in data analysis included familiarizing ourselves with the data, generating initial codes, searching for themes, reviewing and refining themes, defining and naming themes and writing the report.^26^

The use of a postcolonial feminist empowerment model supported the focus on strengths and resilience, while acknowledging the broader sociocultural, economic and political influences that serve to limit youths’ potential for agency. As the study progressed, advisory committee members and youth research assistants encouraged the incorporation of an intersectional lens. Thus, we incorporated an intersectional lens including the delivery of a workshop focussed on intersectionality and considering the intersecting influences on our data. We invited our advisory committee of youths to engage in data analysis by commenting on emerging data patterns, offering additional insights and identifying final relevant themes. The first final product of the youth-led participatory action research was a report co-written by youths and presented to Africa Centre leadership. Before dissemination, we provided participants of both the interviews and conversation cafs with the data for feedback.^27^

***Quality and ethics***

Quality in PAR is defined as ensuring the principles of empowerment, local knowledge development and social action are safeguarded in all aspects of the research process.^23^,^24^ In our study, all research personnel, including the transcriptionist and research assistants, signed a confidentiality agreement. The use of a youth empowerment model grounded in postcolonial feminist theory to guide the PAR allowed us to safeguard research quality by ensuring the research questions were useful to the community, the research findings were grounded in the community’s experiences and the final outcome supported sustainable changes in the community through knowledge dissemination. Furthermore, we incorporated reflexive memos regarding our positioning in the research process over time and consulted with youths regarding their perceptions on inclusion and participation.^28^ Including youths in the analysis phase and writing of the final documents allowed for greater transparency, ownership and legitimacy of findings within the community, and was also crucial for assuring rigor in PAR.^28^

Discussing past experiences of mental health challenges can pose an emotional risk to participants. As noted by Morse et al.,^29^ those who have not had the time to process or resolve a crisis or who have experienced psychic trauma may be highly emotional, and participants may not be aware of upsetting memories until they start discussing them. Hence, ethically important moments may occur during the research.^30^ Research assistants were sensitive to the psychological and emotional needs of the research participants. We offered to stop interviews if participants became emotional. We provided participants in interviews and conversation cafs with a list of resources. Participants were also given an opportunity to debrief at the end of the interviews and conversation cafs to ensure their emotional well-being and referral to appropriate services.

## Results

Our interviews and conversation cafs with Black youths provided rich knowledge at a time when there was a scarcity of information on the mental health of Black youth. Racism and intergenerational gaps in families were the two priority factors identified as contributing to the mental health challenges of Black youth.^8^ Additional factors identified by Black youths include academic stress, microaggression, stigma, financial stress and previous traumatic events. The experience of Black youths illustrated the intersectionality of race, income and other factors. Youths also identified that spirituality, peer support and sense of community contributed positively to their mental health. Youth co-researchers were keen on asking questions about access to culturally appropriate healthcare for Black youth.^3^ Participants identified the need for culturally responsive and safe service provision from both Black and non-Black service providers. Additional barriers to access to services included cost of services and stigma. Mental health services are often located in geographic areas that are not culturally accessible to Black people. These barriers inspired the youths to advocate for the creation of an ideal mental health clinic for Black youth.

***Knowledge mobilization***

We were committed to facilitating a multidirectional flow of knowledge between the research community and knowledge users, including policy makers, decision-makers, community partners, service providers and immigrants. Youths were active participants in the dissemination process. The youths produced a report that was disseminated to diverse stakeholders.^31^ We also held a stakeholder engagement session that was attended by approximately 80 stakeholders including youths, community leaders and members of the Public Health Agency of Canada. The stakeholder event was led by and delivered by the youths. 

In addition, the results of this work were shared by the PI with the Prime Minister of Canada. The results were also shared with the Executive Director of Africa Centre, who used them in collaboration with the Alberta Black Therapists Network to create the first mental health clinic for Black Canadians in Western Canada. The results have also been published in two peer-reviewed papers,^3^,^8^ and several presentations have been made. In our reports, we referred to our participants as “Black youths,” given that all those in our study self-identified as such.

## Conclusion

Our project provided useful knowledge on the mental health of Black youth and informed policy and practice. A strength of this project is that while PAR approaches do not always engage communities from beginning to end, in this case, youths were involved in every aspect of the study and had a strong sense of project ownership. Our prior engagement in the field, consultation with youths and positionality as Black researchers (and two non-Black researchers with strength in participatory research) helped advance the research process. Power relations along the lines of class and age were key challenges that we addressed during the research process. Meetings between one of the PhD student research assistants (BD) and the youths prior to meeting with the research team helped address this challenge. 

As we engaged in the participatory approach, we expected time to be a challenge. However, we were able to collect our data in a period of six months. A challenge we found upon completion of our data collection was that youths who attended multiple cafs often did not complete consent and demographic forms each time they attended. Thus, we had more individuals attend our sessions than the number we recorded. We also had to reconcile gaps between our academic and community priorities. We ensured we did not submit any data for publication prior to submitting a report to our communities. We tackled issues of confidentiality and data security by ensuring youths only had access to the data they collected unless they were engaged in data analysis and needed access to all data. 

Our participant demographics were reflective of youths in the community. Our research assistant demographics (although predominantly female) were also reflective of our participants. Future studies should further examine how to increase gender diversity in research with Black youth. 

All of these efforts produced tangible and meaningful results. We recruited more participants than we had initially proposed for our conversation cafs, built the capacity of youths (many of whom are now graduate students or working in professional jobs) and gathered useful data that changed policy and practice.

## Acknowledgements

We acknowledge funding received from PolicyWise for Children and Families to conduct this research.

## Conflicts of interest

We have no conflicts of interests to disclose, and no financial interest or benefit has arisen from the direct applications of this research.

## Authors’ contributions and statement

BS: conceptualization.

BS, JS:funding acquisition. 

BS, JS, BD, DA:investigation. 

BS, JS, BD, DA:methodology. 

BD, DA: project administration. 

BS: supervision.

BS, AM: writing—original draft.

DA, JS, BD: writing—review and editing. 

The content and views expressed in this article are those of the authors and do not necessarily reflect those of the Government of Canada.
